# Supplementary material for: Are carbon emissions trading and green financial instruments synergistic? -Comprehensive quantitative research based on content analysis
Source: PLoS One. 2024 Mar 7;19(3):e0298601. doi: 10.1371/journal.pone.0298601 (PMC10919601; doi:10.1371/journal.pone.0298601)
Supplement: S1 Data — (ZIP) [file pone.0298601.s001.zip › Data/地级市Z_F工作报告.zip!/─┌├╔╣┼/░═╤σ─╫╢√╩╨/└┤╫╘╣1⁄2╓┌║┼í╛┬φ┐╦╩2╛▌═°í┐_2012.docx]

各位代表：

　　现在，我代表市人民政府，向大会报告工作，请予审议，并请市政协委员和列席会议的同志们提出意见。

　　一、2011年工作简要回顾

　　刚刚过去的一年，在市委的坚强领导下，全市上下团结奋斗，开拓进取，圆满完成了市二届人大四次会议确定的目标任务，实现了“十二五”良好开局。预计地区生产总值完成708亿元，增长约18%；全社会固定资产投资完成634亿元，增长12.2%；社会消费品零售总额完成150亿元，增长约20%；地方财政总收入完成82.5亿元，增长19.8%；城镇居民人均可支配收入完成16580元，增长约15%；农牧民人均纯收入完成9300元，增长约13%。全市经济社会发展承接了“十一五”大好来势，呈现出发展提速、民生改善、社会和谐的可喜局面。

　　（一）结构调整步伐加快，质量效益稳步提升。预计三次产业结构由上年的20:56:24演进为18:58:24。农牧业生产喜获丰收，粮食总产量达到53.5亿斤，创历史新高。新增设施农业4.4万亩，建设规模化养殖小区132个。50个农村土地综合整治工程试点村规划编制完成，其中45个村开工建设。工业经济保持较快增长，预计规模以上工业增加值完成330亿元，增长约20%；实现利润55亿元，增长约40%。盾安多晶硅、中兴能源燃料乙醇、合力机械车辆制造等项目建成投产，神华一期120万吨煤焦化、包钢210万吨煤焦化、新湖50万吨PVC、联邦制药四期和五期等项目加快建设。“双百亿”工程有效实施，园区基础设施进一步完善，20户企业进入内蒙古民营企业100强。服务业稳步发展，巴运物流“甩挂运输”项目完成基础工程，旅游景区建设继续加强，河套农村商业银行挂牌营业。

　　（二）城镇建设快速推进，人居环境持续改善。城建投资突破200亿元，增长24.7%。城市总体规划编制完成并通过自治区批复。棚户区改造有力有序进行，累计签订征收协议面积306万平米，其中临河142万平米。双河区10平方公里核心区建设启动实施，金川大桥开工建设。西区公建项目加快建设，累计28个单位搬迁入驻。临狼、临陕、临磴出口改造全面启动。全市新增园林绿化面积1800亩，永济渠、朔方河、杭后润昇湖、磴口中心湖等湿地保护工程顺利推进，乌前旗林海公园、乌中旗云英广场等公共设施建设各具特色。全市修建主次干道80条，新增天然气入户5600户、集中供热面积280万平米，改扩建供排水管网490公里，城镇面貌继续发生新变化。

　　（三）生态保护力度加大，基础设施建设进展顺利。完成林业生态建设69.3万亩。7208万亩草原列入自治区生态补奖范围，每年可获补奖资金2.93亿元。乌兰布和沙漠刘拐沙头综合治理稳步推进。乌梁素海防凌分洪减灾工程基本完工，投资10.95亿元的水环境治理世行贷款项目启动实施。节水改造、安全饮水等项目建设步伐加快，新增水利投资20.3亿元，是近年来投资最多的一年。铁路、公路等基础设施建设进一步加强，全年新改建公路1024公里，巴彦淖尔机场正式通航。宝音太220千伏、蒙海110千伏等5个输变电工程投入使用。

　　（四）招商引资成效明显，对外开放实现新突破。全年引进国内到位资金357亿元，增长22%。黑猫700万吨煤焦化和200万吨天然气液化、家景450万吨煤焦化、大族3万吨工业硅和40兆瓦光伏发电等一批重大项目签订投资合作协议。与周边地区的产业分工协作不断深化，苏布尔嘎煤田资源配置取得实质性进展，包钢西区建设积极推进。向北开放战略深入实施，神华、中铝等公司对蒙合作项目实现重大突破，甘其毛都口岸原煤进口达到1100万吨，过货量跃居全区第二位。预计进出口总额超过10亿美元，增长近一倍。

　　（五）民生工作扎实开展，人民群众得到更多实惠。各级财政直接用于民生的投入达到68.2亿元，增长39.2%。“15件民生实事”全部完成，“16项民生工程”加快推进。农村牧区劳动力转移就业16.8万人，城镇新增就业1.6万人，城镇登记失业率为3.9%。6个旗县区纳入城乡养老保险试点，6万多名企业退休人员调整提高了养老金。新农合住院报销比例提高到70%，城镇职工和居民医疗保险住院报销比例分别提高到80%、70%。城乡低保月保障标准比上年分别提高35元、41.5元，孤儿、五保对象供养等多项保障标准提高幅度均在12%以上。蒙授高中学生、中等职业学校学生和普通高中家庭经济困难学生免除学费和教科书费。全年减少贫困人口3.5万人，向13.5万名困难群众发放临时价格补贴。新开工各类保障性住房4.46万套。机关事业单位工作人员带薪休假和离退休干部疗养补贴制度全面落实。

　　（六）社会事业协调发展，民主法制建设不断深化。学前教育三年行动计划启动实施，115所学校88万平米校安工程竣工交付使用，河套大学升本取得积极进展。文博中心部分场馆即将开放，新闻大厦搬迁入驻，电影《蓝学校》荣获金鸡百花电影节两项提名，大型情景岩刻史诗《阴山·古歌》即将上演，河套文化影响力日益扩大。医药卫生体制改革走在全区前列，公办基层医疗卫生机构全部落实基本药物制度。新增专利79件、自治区级企业技术中心3家，水环境治理和产业发展院士工作站成立。

　　自觉接受市人大依法监督和政协民主监督，人大代表建议、政协委员提案办结率100%、满意率98%以上。政务公开规范有序进行，“六五”普法全面启动。平安创建工程深入实施，信访工作扎实开展，一批群众反映强烈的突出问题得到解决。基层政权建设继续加强，苏木镇基础设施项目进展顺利，全市增设11个苏木镇获得自治区批准。食品药品安全专项整治成效明显，安全生产形势总体稳定。民族宗教政策全面落实，妇女儿童、双拥共建工作有效推进，被评为“全国实施妇女儿童发展纲要先进集体”、“全区双拥模范城”。

　　各位代表，在“十二五”的开局之年，全市各项工作取得了新的重大进展，朝着赶超崛起的目标迈出了坚实步伐。这是自治区党委、政府和市委正确领导的结果，是历届领导班子团结带领广大干部群众艰苦奋斗、顽强拼搏的结果，也是社会各界同心同德、大力支持的结果。在此，我代表市人民政府，向全市各族干部群众，向所有为巴彦淖尔建设和发展作出贡献的同志们、朋友们，表示衷心的感谢和崇高的敬意！

　　在看到成绩的同时，我们也清醒地认识到存在的困难和问题。主要有：宏观经济形势依然复杂严峻，经济下行和物价上涨压力并存，项目建设要素制约突出；经济总量小，产业发展不充分，立市立县大项目少；中心城区承载能力不强，城镇功能不完善，管理水平亟待提高；基础设施建设相对滞后，生态环境总体上比较脆弱；城乡居民收入增长滞后于经济发展，部分群众生活还比较困难，社会管理和公共服务水平不高；一些工作落实不到位，形式主义、铺张浪费等现象还不同程度存在。我们将以高度负责的态度，认真解决这些问题，努力把各项工作做得更好，绝不辜负全市各族人民的期望和重托！

　　二、2012年的主要工作

　　今年是实施“十二五”规划承上启下的重要一年，也是我市抢抓机遇、发挥优势、赶超崛起的关键一年。政府工作的总体要求是：以科学发展为主题，以转变经济发展方式为主线，全面贯彻落实市第三次党代会和市委三届二次全委（扩大）会议精神，按照“一产做精、二产做强、三产做活、城市做美、生态做优、民生做实”的思路，加快工业化、城镇化、农牧业现代化进程，推进生态和基础设施建设，不断扩大对外开放，加强和创新社会管理，切实保障和改善民生，努力实现经济社会又好又快发展。

　　主要预期目标是：地区生产总值增长17%；地方财政总收入增长20%以上，突破百亿元大关；全社会固定资产投资增长18%以上；社会消费品零售总额增长20%；城镇居民人均可支配收入增长15%，农牧民人均纯收入增长13%；城镇登记失业率控制在4%以内；节能节水减排指标完成自治区下达的目标任务。确定这些目标，基本着眼点是稳中求进、适度超前，体现了赶超发展的新要求和人民群众的新期待，我们一定全力以赴完成，向全市人民交上一份满意的答卷。

　　（一）扩大投资和消费需求，保持经济平稳较快发展

　　促进投资稳定增长。全年完成固定资产投资750亿元，实施亿元以上项目128个。工业推进煤化工、氯碱化工、电力和新能源等项目建设，完成投资335亿元。城建重点加强中心城区和县城建设，完成投资200亿元。水利实施节水改造、水库除险加固、乌梁素海综合治理等重点项目，完成投资20亿元以上。电网建设三盛公220千伏、新华110千伏等22个输变电项目，争取乌后旗、乌中旗2个500千伏风电汇集站早日开工。交通加快锡乌铁路建设，甘泉铁路全线通车，争取临哈高速、甘其毛都口岸至临河一级公路、海流图至甘其毛都口岸公路建设取得实质性进展，推进五原至海流图、青山至乌根高勒、110国道临河绕城线一级公路建设，临河黄河大桥、黄河堤防公路、机场迎宾大道建成通车。增加机场航班航线，保障安全稳定运营。交通完成投资85亿元。

　　持续提升消费水平。发展商贸流通、住宿餐饮等服务业，培育旅游、文化、休闲等消费热点，抑制物价过快上涨。建设B型保税物流园区铁路专用线及监管仓，加强口岸物流、巴运物流等园区建设，完善城乡物流配送体系。高起点开发乌梁素海、纳林湖、乌兰布和分洪区、三盛公水利风景区等旅游项目，建好国家级地质公园，打造精品旅游景区和线路。争取内蒙古银行、交通银行、兴业银行等金融机构在我市设立分支机构，规范民间借贷，防范和化解金融风险。深入实施“万村千乡”市场工程，繁荣农村牧区消费市场。

　　加强对外开放和区域合作。抓好甘其毛都口岸报关报检大楼建设，启用“快捷通”自动通关系统和“五进五出”过煤专用通道，扩建通道卡口至物流园区重载公路，口岸过货量达到1500万吨，力争突破2000万吨。争取实现铜精粉过境，启动实施大型铜资源加工转化项目。推进巴格毛都口岸复关工作，加快编制完善各项规划设计，争取列入国家口岸发展“十二五”规划。加大招商引资力度，继续引进一批大项目、好项目，全年到位资金350亿元以上。加强与周边地区的产业分工协作，在资源配置、产业共育、基础共建等方面取得新进展。

　　（二）加强工业重点项目建设，推进产业结构优化升级

　　发展壮大优势特色产业。农畜产品加工业，实施河套酒业名优酒技改扩建、燕京金川20万吨啤酒、巴美肉羊循环经济产业园等项目，圣牧高科有机奶、嘉源番茄红素等项目建成投产。积极引进有实力的大企业，对面粉、番茄、绒纺等产业进行整合，促进农畜产品加工业持续健康发展。矿山冶金工业，继续开展资源整装勘查，扩大采选规模，发展冶炼和延伸加工。加快推进紫金330万吨铅锌、天宝60万吨铅锌、大中530万吨铁矿等采选项目，争取西部铜业下游产品生产项目开工建设。煤化工，全力实施金牛煤化30万吨合成氨及52万吨尿素项目，神华一期120万吨、包钢210万吨煤焦化及德胜、华油二期天然气液化等项目竣工投产，神华二期360万吨煤焦化和90万吨甲醇项目启动实施。抓紧开展家景煤焦化、盾安乙二醇、黑猫煤焦化及天然气液化等项目前期工作。氯碱化工，加快新湖50万吨PVC项目建设进度，其中100万吨兰炭、50万吨电石建成投产，争取黄河工贸50万吨PVC项目落地建设。电力工业，加速推进临河热电二期、神东中尾煤发电、国网煤电等项目前期工作，支持企业联合建设自备电厂。

　　大力培育新兴产业。推进乌中旗百万千瓦风电基地建设，实施国华三期、国电华北等项目，风电装机容量突破200万千瓦。争取圣泉生物质能源、国电30兆瓦光伏等项目开工建设，兆鑫生物质发电、盾安10兆瓦光伏电站建成投产。抓好盾安二期、汉马二期等多晶硅项目，力争大族3万吨工业硅和40兆瓦光伏发电项目启动实施。加快麦德风机制造、鑫泰物流磁管道等项目建设，中科宇能风电叶片、合力机械异型钢等项目竣工投产。联邦制药四期和五期项目年内试车。

　　加快实施“双百亿工程”。扶持重点企业提速发展，力争2013年紫金、联邦制药、西部铜业营业收入率先超过百亿元。培育大中、盾安、河套酒业等一批高成长性企业，形成梯次强企方阵。进一步明确工业园区功能定位，加大基础设施建设力度，在土地收储、项目引进等方面取得新进展，甘其毛都口岸加工园区、乌后旗工业园区营业收入超过百亿元。支持巴彦淖尔经济开发区创建国家级开发区。认真落实促进中小企业发展的政策措施，帮助中小企业解决实际困难和问题，建好临河中小企业创业园。

　　（三）做好“三农三牧”工作，促进农牧民持续增收

　　加快发展现代农牧业。扶持优质小麦种植，粮食总产量稳定在50亿斤以上。市本级财政安排专项资金，支持设施农业和规模化养殖业发展，新增设施农业5万亩，新建、改扩建280个高标准规模化养殖小区和家庭生态牧场，牲畜饲养量突破2000万头（只）。推进农牧业产业化项目建设，围绕脱水蔬菜、番茄、乳肉等优势产业，加快建设一批集中连片农畜产品生产基地，新增土地流转50万亩。大力实施品牌战略，提升巴美肉羊、河套番茄、葵花籽、面粉、瓜果等特色产品的知名度。

　　改善农村牧区生产生活条件。实施农村土地综合整治工程，在加快建设50个试点村的基础上，继续高标准规划建设50至100个新农村示范点，争取将主要交通干道沿线、县城周边的村庄基本整治完毕。市本级财政安排2060万元专项资金，扩大“一事一议”财政奖补试点范围。抓好农田水利、草牧场和饲草料基地建设，推进中低产田改造、井灌区高标准农田基本建设等重点工程。新建农村牧区油路600公里，完成村镇绿化40个，新增户用沼气5000户，解决3.5万人饮水安全问题，实施2万户农村牧区危旧房改造，为贫困农牧民安装卫星接收系统1万套。

　　强化农牧业社会化服务体系建设。推进“种子工程”，加快良种繁育，大力推广优质高产新品种。扩大农牧业政策性保险覆盖面，减轻农牧民受灾损失。抓好科技、气象、金融等服务，提升农牧业机械化水平。完善动植物疫病防控体系，加强农产品质量安全监管，新增无公害农产品、绿色和有机食品认证23个。发展壮大各类专业合作组织，提高农牧民组织化程度。

　　（四）加快建设生态宜居城市，切实提升规划建设管理水平

　　加大棚户区改造力度。完成180万平米改造任务，其中临河100万平米，改善1.82万户居民的居住条件和生活环境，实现三年基本完成棚户区改造的既定目标。配建经济适用住房1470套，廉租房4755套，公共租赁房1508套，限价商品房7795套。坚持依法征收，全面落实“三公”、“两监督”等措施，千方百计做好房屋征收工作。完善回迁安置制度，从根本上解决回迁房质量不高、建设进度缓慢等问题。

　　推进双河区开发建设。加快城市设计编制进度，抓好土地征收报批工作。金川大桥10月底前竣工通车，实现主城区与双河区快速连通。高标准建设滨河景观大道，实施金川南路、云中大街等12条主次干道建设，配套给排水、供电等基础设施。启动河套大学新校区、党校新校区、体育馆等建设工程，争取部分建筑物主体封顶，拉开10平方公里核心区基本框架，打造城市建设新亮点。

　　加强湿地保护恢复和园林绿化。临河重点实施青春湖、水源湖、镜湖等湿地保护与恢复工程，启动总干渠先锋桥至金川大桥段水系景观建设，抓好先锋桥东西各15公里衬砌和绿化。加快永济渠、永刚渠、北边渠、金川河水系景观建设进度，完成朔方河河道、桥梁、绿化工程。各旗县依托城镇周边湖泊湿地、灌排渠系、沟口河槽，通过生态补水、引水入城、开挖人工河湖等措施，因地制宜规划建设水系景观。加大园林绿化力度，实施水系绿化、公园绿化、广场绿化和主要街路植绿补绿工程，推进临河森林公园千亩生态园、北郊公园及县城园林景观建设，全年新增绿化面积2300亩。

　　完善城镇基础设施。加大旗县区所在地出口综合整治力度，彻底改变脏乱差现象，重点抓好临陕、临狼、临磴等出口改造，配套道路景观、绿化美化和专业市场。推进河套大街综合改造，争取项目建设尽快全线铺开。实施城镇电缆入地工程，启动临河第三自来水厂二期和第四自来水厂项目，加快万丰变电站至临河西区电力通道建设。全年新建改造道路45条，铺设各类管网390公里，新增集中供热面积300万平米、天然气入户1.2万户，新建垃圾转运站20座、水冲式公厕25座。高标准实施建筑立面改造和亮化美化工程，对主要街路、背街巷道、重要节点进行综合治理，实现路平、灯明、街绿、城美。

　　提高城镇管理水平。严格执行城镇规划，切实维护规划的严肃性、权威性。强化执法巡查，严厉打击私搭乱建行为，坚决拆除违章建筑。深入开展城镇卫生大整治，营造干净整洁的市容环境。加强交通管理，优化公交线路，增加营运车次，下大力气解决交通拥堵问题。整顿马路市场、流动摊贩，做到划行归市、规范管理。强化法制和社会公德教育，提升市民文明素质。

　　（五）坚持美丽与发展共赢，扎实推进秀美巴彦淖尔建设

　　加强生态建设和保护。继续实施天然林保护、退耕还林等重点工程，完成林业生态建设100万亩。严格执行草畜平衡、禁牧休牧轮牧和基本草原保护制度，全面落实草原生态补奖政策，年内减少载畜量52万头（只），新增草原禁牧面积2100万亩以上。加强乌兰布和沙漠防风固沙林带建设，启动刘拐沙头综合治理四期工程，科学发展沙草产业。推进乌梁素海世行贷款水环境治理项目，实施网格水道、人工湿地等工程。加大河套平原造林绿化力度，高标准推进环城、环镇、环村、环路绿化，打造黄河堤防公路造林、机场专用公路绿化等精品示范工程。抓好灌区绿化、盐碱荒地绿化和农田林网建设，尽快使所有宜林地全部绿化，展现绿色河套新形象。

　　做好资源节约和环境保护工作。推动建筑、交通和公共机构节能，完成50万平米建筑节能改造。加强钢铁、水泥等非电行业脱硫脱硝设施建设，实施临河、前旗2个电厂脱硫增容、金牛煤电脱硝等项目。加快乌后旗等3个工业园区污水处理厂建设进度，启动实施临河第二污水处理厂。严把项目准入关，坚决淘汰落后产能。大力推进秸秆、粉煤灰、煤矸石等资源综合利用，培育一批循环经济示范企业和园区。严格保护耕地，切实抓好土地矿产卫片执法检查工作。集约利用水资源，严禁抽取地下水发展高耗水产业。在全社会牢固树立生态文明理念，全力保护好河套这方水土，让巴彦淖尔林更茂、地更绿、天更蓝、水更清！

　　（六）继续加大民生投入，切实保障和改善民生

　　多渠道增加城乡居民收入。推行企业工资集体协商，完善最低工资制度和工资正常增长、支付保障机制，规范公务员津补贴，推进事业单位绩效工资制度改革，提高城镇居民收入水平。拓宽农牧民增收渠道，认真落实各项惠农惠牧政策，引导农村牧区富余劳动力向二、三产业转移，不断增加农牧民经营性、工资性、转移性收入，农牧民收入增幅继续高于全区平均水平。

　　千方百计扩大就业。大力发展劳动密集型产业、服务业、中小企业和民营经济，多渠道开发就业岗位，加强公共就业创业服务体系建设，城镇新增就业1.2万人，农村牧区劳动力转移就业16万人。继续实施人才储备工程，落实好“三支一扶”等高校毕业生服务基层计划。帮助2000名城镇就业困难人员实现就业，保持“零就业家庭”、“零转移家庭”动态消零。帮助企业解决用工难问题，切实维护农民工合法权益。积极创建国家级创业型城市。

　　全面提高社会保障水平。今年市本级财政安排社会保障专项支出11208万元，较上年增加1562万元，向上级争取社会保障与就业转移支付资金4亿元。实现城乡养老、医疗保险全覆盖，将农民工全部纳入工伤保险范围。提高城镇职工和居民医疗保险住院报销比例。新农合实现市级统筹，政策范围内住院报销比例稳定在70%以上。继续提高企业退休人员基本养老金，城乡低保保障标准月人均分别提高51元、30元，五保对象集中和分散供养标准年人均分别提高900元、500元，孤儿集中和分散供养标准月人均分别提高192元、160元，城镇“三无人员”集中和分散供养标准月人均分别提高129元、80元。加强民政福利园区、老年社区等社会福利设施建设。积极争取国家和自治区扶贫资金，市本级财政安排扶贫专项资金1200万元，重点实施集中连片特困地区、整村推进、易地移民搬迁扶贫“三大工程”，减少贫困人口3万人。

　　各位代表，民生连着民心，民心凝聚民力。我们一定切实解决好群众最关心最直接最现实的利益问题，今年各级财政用于民生的投入达到40%以上，让群众更多更好地分享改革发展成果！

　　（七）大力发展社会事业，维护社会和谐稳定

　　坚持教育优先发展。今年市本级财政安排教育专项支出8811万元，较上年增加3471万元。新建、改扩建23所幼儿园，全面完成校安工程三年规划任务，推进义务教育标准化建设，义务教育阶段残疾儿童就学全免费。实现高中阶段免费教育，提前一年完成目标。抓好市蒙古族学校搬迁和职业学院筹建工作。支持河套大学建设和发展，争取实现升本目标。深入实施名校长、名教师、名学校建设工程，全力打造教育品牌。多层次资助困难家庭学生，确保不让一个学生因贫辍学。

　　推进民族文化大市建设。今年市本级财政安排文化专项支出1380万元。深化文化体制改革，加快发展文化产业，组建巴彦淖尔报社传媒集团。文博中心投入使用，广电传媒中心主体完工，启动鸿雁大剧院建设。新建28个苏木镇综合文化站，配套完善150个嘎查村和社区文体活动室。深入挖掘河套文化内涵，设立河套文化创作生产基金、“河套文化创作奖”，集中力量推出一批精品力作，阴山岩刻进入“国家世界文化遗产预备名单”。广泛开展精神文明创建活动，全面实施“全民健身计划”，办好全市第二届运动会和首届残疾人运动会。

　　加快发展医药卫生事业。今年市本级财政安排医药卫生专项支出11621万元，较上年增加1317万元。深化医药卫生体制改革，基本药物制度扩大到嘎查村卫生室，推进县级公立医院体制改革。市医院新址开工建设，蒙医院、精神病院改建和4所旗县医院续建项目完工并投入使用，实施10所苏木镇卫生院和社区卫生服务中心、50所嘎查村卫生室新改扩建工程。全面推行食品药品安全网格化管理。稳定适度低生育水平，提高出生人口素质，推进“一杯奶”生育关怀行动。

　　提高科技创新能力。今年市本级财政安排科技专项支出1030万元，较上年增加100万元。加强科技服务平台建设，建成自治区级科技企业孵化器。积极培育国家级高新技术企业，争取新建3个以上自治区级企业技术中心。启动1至2家产业技术创新战略联盟，高标准建设农牧业科技示范园区。加快培育和引进科技人才，用好市级科技创新基金，支持重点科研项目和科技成果转化。

　　加强和创新社会管理。强化基层组织社会管理服务职能，做好新设苏木镇区划勘定、机构设置等工作，实施一批苏木镇基础设施项目。加强城镇社区建设，临河区建设2个、其他旗县建设1个高标准社区服务中心，用三年时间基本完成社区建设达标任务。深入开展领导干部大接访活动，加大社会矛盾排查化解力度。完善社会治安防控体系，加快视频监控和联网报警系统建设，严厉打击各类违法犯罪行为。强化安全生产监管，坚决遏制重特大事故发生。全面落实党的民族宗教政策，推进稳边固边兴边工程，巩固社会稳定、民族团结、边疆安宁的局面。

　　（八）加强政府自身建设，打造人民满意政府

　　全面推进依法行政。严格依照法定权限和程序履行职责，保障各项工作依法规范开展。坚决执行市委的决策部署，主动接受人大及其常委会的法律监督、政协的民主监督，做好人大代表建议、议案和政协委员提案办理工作。广泛听取各民主党派、工商联和无党派人士意见，支持工会、共青团、妇联等人民团体发挥桥梁纽带作用。完善科学民主决策机制，提高决策效率和水平。

　　加快转变政府职能。按照责权利相统一的原则，理顺行政管理体制。深化政务公开工作，能公开的事项都要及时全面公开。推进市政务服务中心规范化建设，力争部门100%进厅、项目100%到位、环节100%监察。切实优化投资环境，下大力气解决办事拖沓、推诿扯皮、吃拿卡要等问题。充分发挥“三问”平台作用，解决好群众关心的热点难点问题。健全考评机制，加大督查力度，形成工作有抓手、绩效有评估、过失必追究的运行体系，确保政府工作高效运转。

　　切实改进工作作风。大力践行“一线工作法”，做到问题在一线发现，矛盾在一线解决，业绩在一线创造，成效在一线检验。积极倡导雷厉风行、真抓实干的作风，做到言必信，行必果，一抓到底，务求实效。对项目建设、民生工程等事关全局的重点工作，细化目标任务，明确时限要求，调动各方力量加快推进。切实减少会议和文件，开短会、讲短话、发短文，腾出更多时间抓落实。

　　大力加强廉政建设。全面落实党风廉政建设责任制，严格执行廉洁从政各项规定，旗帜鲜明地推进反腐倡廉建设。加强监察和审计监督，坚决查处各类违纪违法案件。坚持勤俭节约，反对铺张浪费，继续压缩一般性支出，各级党政机关公务接待、出国经费、车辆购置等支出保持零增长。政府全体工作人员要清白做人，干净做事，扬正气、树新风、谋发展、促和谐。

　　各位代表，新的一年孕育新的希望，新的起点蕴含新的机遇。让我们紧密团结起来，在市委的正确领导下，以昂扬向上的精神、知难而进的勇气、争创一流的决心，抢抓机遇，奋力拼搏，扎实工作，把巴彦淖尔建设得更加繁荣富裕、和谐美好，以优异成绩迎接党的十八大胜利召开！
